# Missed nursing care in acute care hospital settings in low-income and middle-income countries: a systematic review

**DOI:** 10.1186/s12960-023-00807-7

**Published:** 2023-03-14

**Authors:** Abdulazeez Imam, Sopuruchukwu Obiesie, David Gathara, Jalemba Aluvaala, Michuki Maina, Mike English

**Affiliations:** 1 KEMRI-Wellcome Trust Research Programme, Nairobi, Kenya; 2 Health Systems Collaborative, Nuffield Department of Medicine, University of Oxford, S Parks Rd, Oxford, OX1 3SY UK; 3 Centre for Evidence Based Intervention, Department of Social Policy and Intervention, University of Oxford, Oxford, UK; 4 MARCH Centre, London School of Hygiene and Tropical Medicine, London, UK; 5 Department of Paediatrics, University of Nairobi, Nairobi, Kenya

**Keywords:** Quality of care, Developing countries, Nurses, Patient safety, Omission of care, Rationing care, Unmet patient needs, Missed care, Care left undone

## Abstract

**Background:**

Missed nursing care undermines nursing standards of care and minimising this phenomenon is crucial to maintaining adequate patient safety and the quality of patient care. The concept is a neglected aspect of human resource for health thinking, and it remains understudied in low-income and middle-income country (LMIC) settings which have 90% of the global nursing workforce shortages. Our objective in this review was to document the prevalence of missed nursing care in LMIC, identify the categories of nursing care that are most missed and summarise the reasons for this.

**Methods:**

We conducted a systematic review searching Medline, Embase, Global Health, WHO Global index medicus and CINAHL from their inception up until August 2021. Publications were included if they were conducted in an LMIC and reported on any combination of categories, reasons and factors associated with missed nursing care within in-patient settings. We assessed the quality of studies using the Newcastle Ottawa Scale.

**Results:**

Thirty-one studies met our inclusion criteria. These studies were mainly cross-sectional, from upper middle-income settings and mostly relied on nurses’ self-report of missed nursing care. The measurement tools used, and their reporting were inconsistent across the literature. Nursing care most frequently missed were non-clinical nursing activities including those of comfort and communication. Inadequate personnel numbers were the most important reasons given for missed care.

**Conclusions:**

Missed nursing care is reported for all key nursing task areas threatening care quality and safety. Data suggest nurses prioritise technical activities with more non-clinical activities missed, this undermines holistic nursing care. Improving staffing levels seems a key intervention potentially including sharing of less skilled activities. More research on missed nursing care and interventions to tackle it to improve quality and safety is needed in LMIC.

*PROSPERO registration number:* CRD42021286897.

**Supplementary Information:**

The online version contains supplementary material available at 10.1186/s12960-023-00807-7.

## Background

Kalisch et al. define missed nursing care as patient care that is wholly or partially missed or delayed during the conduct of nursing duties [1, 2]. The authors developed a framework to understand the concept based on the Donabedian structure–process–outcome model [1, 3]. Essentially, this framework describes influences on nurses’ internal decision-making process to prioritise some aspects of patient care over others due to increased pressures from structural aspects of their work environments, such as patient care demands or available labour and material resources [1]. This is now supported by evidence which suggests low nurse staffing and high patient load are associated with missed nursing care [4].

Missed nursing care has been described in the literature using some other terms including ‘task left undone’, ‘unmet needs’ or implicit rationing [4]. It has significant relevance to patient safety and quality of care in acute hospital care settings and is associated with negative patient care outcomes, such as medication administration errors, hospital acquired infections and patient mortality [5–9]. Increased levels of missed nursing care have also been associated with decreased patient satisfaction and poor nurse-reported hospital quality of care ratings [10].

Reviews of missed nursing care have approached the concept from a variety of angles. They have summarised interventions aimed at minimising missed nursing care [11], examined specific or multiple factors associated with the concept [4, 12–16], and reviewed frameworks and instruments used to measure it [13]. Others have summarised the missed nursing care literature from a patient’s perspective [17], and examined the evidence relating missed nursing care to specific patient care outcomes [10, 12, 18]. Researchers have also integrated the findings from multiple reviews into an overview of reviews [19]. Common to these reviews is that the summarised literature on missed nursing care largely come from high-income countries. This is likely to be because tools to measure missed care originate from high-income countries with validation of these tools and the conduct of research in low and middle-income countries (LMIC) following later. It is likely, however, that missed nursing care affects nursing throughout the world but is under-reported and understudied in LMIC settings. A synthesis of the available literature from these countries would provide crucial information for researchers and policymakers.

LMIC are heterogenous in terms of their human and material resources in health care, although LMIC hospitals typically have poorer staffing and equipment compared to high-income countries [20]. This reflects that 90% of global nursing shortages occur in LMIC [21]. As nurse staffing levels are strongly associated with missed nursing care [4], It is thus possible the frequency, or type of care that is missed might differ in LMIC. There are now adapted versions of some existing tools to measure missed nursing care and translations to local languages in some LMIC [22–24]. For example, a commonly used tool, the Missed nursing care survey (MISSCARE) now has an adapted Brazilian and Chinese version [25, 26]. These more recent versions differ subtly from the original MISSCARE in terms of their content and the number of nursing activities they assess. In addition, there are now some examples of tools developed in LMIC which assess context-specific nursing activities [27]. These have led to more research being conducted in LMIC settings in recent times. Integrating such data in a systematic review is likely to provide deeper understanding of the concept in LMIC, contribute to a broader and more international understanding of missed nursing care and might guide future research to influence staffing policies in such settings.

### Aim and objectives

The aim of this systematic review is to document the prevalence and categories of the most frequently missed nursing care activities in LMIC and document the associated factors and reasons for this. Our specific objectives include:

1. To determine the prevalence of missed nursing care and the categories of nursing care that are most frequently missed in acute hospital settings in LMIC
2. To document the factors associated with and reasons for missed nursing care in LMIC settings.

## Methods

### Research design

This systematic review was conducted and reported using the PRISMA guidance [28]. Our review protocol was registered with the International Prospective Register of Systematic Reviews (PROSPERO), Registration number CRD42021286897 and was also published [29].

### Data sources and search strategy

To identify eligible primary papers for our review, we conducted a systematic search of 5 electronic databases: Medline, Embase, Global Health, WHO Global index medicus and Cumulative Index to Nursing and Allied Health Literature (CINAHL) from their inception up until August 2021. No date restriction filters were applied to this search. We also searched references of our included papers and conducted forward searching in Scopus. Our search strategy and search terms are detailed in the Additional file 1**.**

### Selection of primary papers

#### Screening

We managed our references and performed deduplication using the Zotero reference software [30], and exported the final set of articles for screening in Rayyan [31]. Two reviewers, AI and SO independently screened article titles and abstracts for eligibility and selected potentially eligible papers for full-text screening before agreeing on a final set of papers to include in the review.

### Inclusion and exclusion criteria

Pre-specified eligibility criteria were any quantitative study which reported on any combination of categories, reasons and factors associated with missed nursing care within in-patient settings in a LMIC setting and which was published in English [29].

#### Population

We included original studies which focused on patient care that was missed by staff nurses or midwives. We excluded studies that examined missed care among other cadres of healthcare professionals including nurse assistants [29].

#### Exposures

Our exposures for this review were the categories, reasons and risk factors associated with missed nursing care. Risk factors for missed nursing care are patient, nurse, or hospital-level factors for which an association was investigated with missed nursing care. Reasons for missed nursing care are nurse reported reasons for why missed care occurred.

#### Outcome

Our outcome for this review is missed nursing care. We considered papers which used other synonyms of missed nursing care, for example, omission of care, unmet nursing needs and implicit rationing of nursing care. We excluded studies which reported on medication errors among nurses as these are errors associated with commission, unlike missed nursing care which arises from omission.

#### Setting

We focused our review on acute care hospital settings, as the current evidence for missed nursing care is largely described in these settings [4]. We excluded papers from ambulatory or community care, for example, missed nursing care in nursing homes. We also considered only studies conducted in LMIC. The definition of LMIC was operationalised using the World Bank country and lending group classification system which classifies countries into low-income, low–middle-income and upper-middle-income economies based on gross national income per capita [32]. For multi-country studies conducted across both HIC and LMIC settings, we included these if we were able to separately extract LMIC results from the papers.

### Quality assessment

We evaluated each paper using the Newcastle–Ottawa Scale [33], which is widely used for non-randomised studies and there is an adapted version for cross-sectional studies [34]. It comprises 7 questions with a maximum score of 10 and these are across three main categories; sample selection, comparability of study groups and outcome assessments. [34] We classified the studies into high quality [7–10 points], medium quality (4–6 points) and poor quality (0–3 points). Both AI and SO conducted independent risk of bias assessments and managed disagreements through discussion.

### Data extraction

AI and SO independently extracted data from the final set of papers. This included the first author surname and year of publication, the study objective and design, the country and setting, where the research was conducted, the study population, sample size, type of exposure/intervention studies and the instrument used to measure missed nursing care.

### Data synthesis

The findings of this systematic review are presented using tables and in narrative synthesis form. We extracted the overall estimate of missed nursing care (median Likert score or overall percentage of care missed) from the individual papers.

To determine the categories of nursing care that were most frequently missed, we used a method similar to that in a previously published review by Griffiths et al. [4]*.* We rank ordered nursing activities from the least to most missed within specific reports and studies using either the MISSCARE or MISSCARE Brasil tools (Table 1). These were the two most used tools and are broadly similar. Other tools were employed in 1 or 2 studies only (Table 1). We only combined studies which reported complete information, for example, for a study to be included in our analysis, the researchers would have needed to report on all 24 nursing activities of the MISSCARE tool. We calculated a median rank across all studies using the MISSCARE or MISSCARE Brasil survey tools and determined the relative frequency of nursing activities missed by ordering the cross-study median ranks from the least to most missed nursing activity. Although this meant focusing on a subset of reports, it was not practical to combine data across primary studies which used different tools as these varied in length and type of nursing activities they examined (Table 1).

**Table 1:** Summary of instruments used to measure missed nursing care in LMIC settings

Instrument (Original reference for tool)	Level of measurement of missed nursing care	Brief tool description	Adaptations	Number of questions	Scale/score category	Studies in the review employing specific tool (references in footnote)
Basel Extent of Rationing of Nursing Care—Revised (BERNCA-R) [38]	Nurse self-report	Scale consists of 32 nursing activities, nurses report on the degree to which they were unable to carry these out in their preceding week of duty Activities are broadly divided into 5 domains of care: – Activity of Daily livings, e.g., Bathing, bed linen change – Caring-support – Rehabilitation-instruction-Education – Monitoring-Safety – Documentation	Revised from the BERNCA which was adapted from the International Hospital Outcome Study	32	5-point Likert scale that measures the frequency with which care was missed: 0 = not required (i.e., rationing of nursing activity was not required in the last week) 1 = never 2 = rarely 3 = sometimes 4 = often	a, b
MISSCARE [39]	Nurse self-report	Two parts (A and B). Part A is designed to measure missed nursing care and consists of a list of nursing activities; nurses are asked to report if they missed these on their previous shifts Part B is designed to measure perceived nurse reasons for missed nursing care around 3 domains—Labour, material, and communication/teamwork	Not applicable	Part A—24 questions Part B—17 questions	5-point Likert scale to measure the frequency of missed nursing care: 1 Never missed (i.e., nurse activity being measured is never missed) 2 Rarely 3 Occasionally 4 frequently 5 Always missed	c, d, e, f, g, h, i, j, k, l, m, n, o
MISSCARE-Brasil [25]	Nurse self-report	Same as MISSCARE tool	Addition of a few questions to reflect the Brazilian context	Part A—28 questions Part B-28 questions	Same as MISSCARE tool	p, q, r, s, t, u
MISSCARE-Chinese [26]	Nurse self-report	Same as MISSCARE tool	Some original MISSCARE questions modified with a few additional questions to reflect the Chinese context	Part A—29 questions Part B—22 questions	Same as MISSCARE tool	v
MISSCARE (modified by maternal health experts) [40]	Nurse self-report	A modification of the MISSCARE tool my maternal health experts Contains questions covering timely cervical examinations and labour support to fit nursing activities in an Obstetrics and Gynaecology unit	Adapted specifically to assess missed care in Obstetrics and Gynaecology	26 questions	Same as MISSCARE tool	w
MISSCARE-PU [41]	Nurse self-report	Abridged version of MISSCARE tools with some modified questions and additional questions to cover aspects of pressure ulcers management	Adapted specifically to assess missed care in nurses’ management of pressure ulcers	13 questions	Same as MISSCARE tool	x
Missed Nursing Care Scale (MNCS)	Nurse self-report	Questionnaire covers 12 essential nursing tasks which were left undone on the most recent nursing shift	Not applicable	12 questions	4-point Likert scale measuring the degree to which nursing activities were left undone: 0 never 1 rarely 2 occasionally 3 frequently	y, z
Nursing Care Index (NCI) [27]	Direct observation of patients	Structured tool used by a bedside observer to collect data around care delivered to a newborn. Covers domains, such as routine newborn care, vital sign monitoring and medications	Not applicable	Not applicable	Observed care are summed up and expressed as a proportion of explicitly defined expected care to derive a patient-level aggregate score of care	aa
RN4Cast Questionnaire [9]	Nurse self-report	Questionnaire covering a list of 13 necessary nursing activities to which nurses are asked to identify which were left undone in their most recent shifts because of time constraints. Activities measured include those related to clinical care, patient care planning and communication	Adapted from the from International Hospital Outcome Study	13 questions	Binary—care is missed or not missed, and results are reported as percentage of specific care that is missed	ab
Unnamed tool [42]	Nurse self-report	Questionnaire covers a list of 15 nursing activities including clinical, planning patient education and counselling	Not applicable	15 questions	Binary—care is missed or not missed, and results are reported as percentage of specific care that is missed	ac

a Assaye et al. [23], b Zhu et al. [37], c Arslan et al. [43], d Nahasaram et al. [57], e Al-Faouri et al. [24], f Hammad et al. [49], g Chegini et al. [46], h Bacaksiz et al. [44], i Saqer et al. [22], j Hernández-Cruz et al. [50], k Kalisch et al. [51], l Moreno-Monsiváis et al. [56], m Ghezeljeh et al. [48], n Taskiran et al. [61], o Grajales et al. [62], p Moura et al. [55], q Lima et al. [54], r Dutra et al. [47], s Silva et al. [60], t Pereira Lima Silva et al. [59], u Siqueira et al. [25], v Du et al. [26], w Haftu et al. [40], x Valles et al. [41], y Labrague et al. [52], z Labrague et al. [53] aa Gathara et al. [27], ab Nantsupawat et al. [58], ac John et al. [42]

To identify whether there was a pattern across activities that were missed, we used the six domains of nursing care described by the American Nurses Association (ANA) to categorise missed nursing activities in all reports [35]. This allowed broader semi-quantitative comparisons across reports using tools which differed in content. These domains include patient assessment, provision of emotional support, medical needs, physical needs, planning and teaching. We added a 7th category—undefined—to identify activities that did not fit into any of these 6 domains [35]. Nursing activity categorisations were performed independently by 5 reviewers (AI, ME, DG, MM, AJ) and consensus was achieved when 4 out of 5 of the reviewers agreed on a classification.

We extracted data on nurses’ self-reported reasons for missed care from studies that employed the MISSCARE tool (This collects data on pre-defined reasons for missed nursing care). We employed a similar ranking method as we used above to determine the most important reasons for missed nursing care across studies. We also extracted factors associated with missed nursing care reported by individual studies. We determined what proportion of studies reported these to be statistically significant using a bubble plot and semi-quantitatively determined how the risk of bias assessments affected variable significance.

## Results

### Search results

**Fig. 1:**
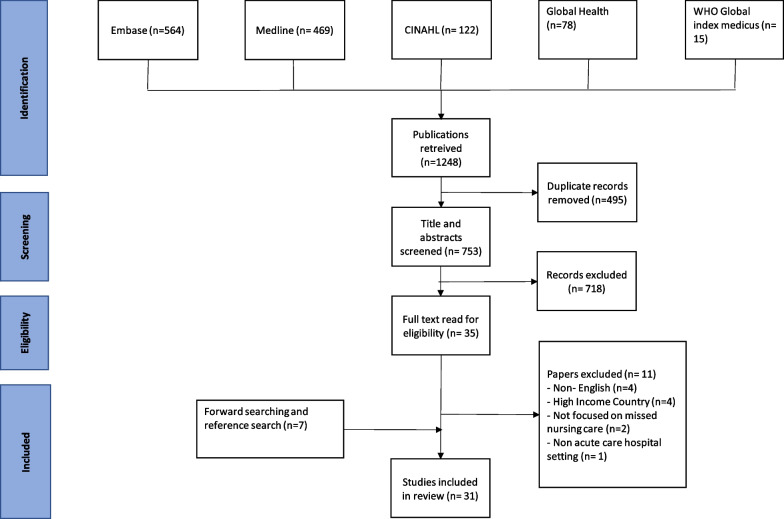
PRISMA diagram

From 1248 articles from our initial search of 5 databases, we excluded 495 duplicate articles and screened the title and abstract of 753 remaining articles. From these, we identified 35 eligible articles for full-text screening and included 24 of these. We identified 7 additional papers from reference searches of the included papers and forward searching (Additional file 1). In total we include 31 papers in our final synthesis (Table 2). The PRISMA flow chart in Fig. 1 provides a summary of our screening process, while Additional file 2 contains a list of our excluded papers and reasons for their exclusion.

**Table 2:** Overview of the included systematic reviews showing the review objective and geographical locations, where the reviews primary studies were conducted

First author (year)	Study location	Study design	Study setting	Sample population and size	Exposure for missed nursing care studied	Missed nursing care tool	Prevalence of missed nursing care-reported median Likert score (scale)/%
Al-Faouri et al. (2021) [24]	Jordan	Cross-sectional	3 hospitals (public, private and university) in Jordan	300 Nurses	Factors and reasons	MISSCARE (Arabic translated)	**2.16 (1.00–5.00)**
Arslan et al. (2021) [43]	Turkey	Cross-sectional	Surgical, Medicine, and Intensive Care units of 3 tertiary hospitals	233 Nurses	Ethical leadership	MISSCARE (Turkish translated)	**1.41 (1.00–4.00)**
Assaye et al. (2022) [23]	Ethiopia	Cross-sectional	Medical and surgical units in two (public and private) hospitals	74 and 80 nurses (2 timepoints), 517 patients	Factors	BERNCA-R (Translated to Amharic)	**2.04 (1.00–4.00)**
Bacaksiz et al. (2020) [44]	Turkey	Cross-sectional	25 private hospitals	897 Nurses	Factors and reasons	MISSCARE (Turkish translation)	**1.39 (1.00–5.00)**
Bekker et al. (2015) [45]	South Africa	Cross-sectional	60 medical and surgical units in private hospitals and public hospitals	1166 nurses	Non-nursing tasks and missed care	MNCS	**Not reported**
Chegini et al. (2020) [46]	Iran	Cross-sectional	Medical and surgical unit of 8 public and private hospitals	215 Nurses	Factors and reasons	MISSCARE (translated to Persian)	**2.57 (1.00–5.00)/72.1%**
Du et al. (2020) [26]	China	Cross-sectional	34 secondary and tertiary hospitals	6158 Nurses	Factors and reasons	MISSCARE-Chinese	**2.98 (1.00–5.00)**
Dutra et al. (2019) [47]	Brazil	Cross-sectional	Adult hospitalization units for clinical and surgical treatment of a single tertiary (teaching) hospital	58 Nurses and nursing technicians	Types and reasons	MISSCARE-Brasil	**74.1%**
Gathara et al. (2020) [27]	Kenya	Cross-sectional	Six health facilities in Kenya. (Public, private and mission hospitals)	216 Newborn infants	Prevalence and factors	Nursing Care Index	86%*
Ghezeljeh et al. (2020) [48]	Iran	Cross-sectional	Emergency departments in educational medical centres affiliated to a university (tertiary)	213 Nurses	Factors	MISSCARE (Persian translation)	**Not reported**
Haftu et al. (2019) [40]	Ethiopia	Cross-sectional	Obstetrics and gynaecologic units in 8 general hospitals	401 Nurses and midwives	Factors and reasons	MISSCARE (modified by maternal health experts)	**74.6%**
Hammad et al. (2021) [49]	Egypt	Cross-sectional	50 units at a single tertiary Hospital	553 Nurses	Factors and reasons	MISSCARE (Arabic translated)	**2.26 (1.00–5.00)**
Hernández-Cruz et al. (2017) [50]	Mexico	Cross-sectional	A single private hospital	71 Hospital nurses	Factors	MISSCARE	**Not reported**
John et al. (2016) [42]	Nigeria	Multi-method (Cross-sectional, Before and after study design (interventional)	Medical, surgical, Obstetrics and Gynaecology units of 4 hospitals providing direct adult care (2 tertiary and 2 secondary-level)	186 nurses and 120 patients/relatives	Prevalence and frequency Effect of a 4-week capacity building intervention for nurses	Unnamed tool	**83.9%**
Kalisch et al. (2013) [51]	Lebanon and US	Cross-sectional	Medical-surgical unit, intermediate unit, and ICU in a single tertiary (teaching) hospital	114 Nurses	Factors and reasons	MISSCARE	**1.21 (1.00–4.00)**
Kalisch et al. (2020) [36]	Egypt	Before and after study design	Single paediatric nephrology unit at a tertiary (teaching) hospital	28 Staff nurses	MISSCARE orientation program	Missed Nursing Care Observational Checklist,	**2.31 (1.00–3.00)**
Labrague et al. (2020) [52]	Philippines	Cross-sectional	6 hospitals in the Philippines	549 Nurses	Nurse caring behaviours	MNCS	**1.21 (1.00–4.00)**
Labrague et al. (2022) [53]	Philippines	Cross-sectional	14 hospitals (7 government 7 private hospitals at various levels of healthcare)	295 nurses	Factors	MNCS	**Not reported**
Lima et al. (2020) [54]	Brazil	Cross-sectional	Ten hospitalization units of a single public tertiary (teaching) hospital	267 nurses, technicians, and auxiliaries	Prevalence and reason	MISSCARE-Brasil	**Not reported**
Moura et al. (2020) [55]	Brazil	Longitudinal Interventional	4 In-patient units at a single tertiary (university) hospital	96 Nurses	Primary Care Nursing Model	MISSCARE Brasil	**Not reported**
Moreno-Monsiváis et al. (2015) [56]	Mexico	Cross-sectional	Medical and surgical units of a single private hospital	160 Nurses and 160 private patients	Factors	MISSCARE	**Not reported**
Nahasaram et al. (2021) [57]	Malaysia	Cross-sectional	Medical and surgical unit of a large tertiary hospital	364 Nurses	Factors and reasons	MISSCARE (Malay translated)	**1.88 (1.00–5.00)**
Nantsupawat et al. (2022) [58]	Thailand	Cross-sectional	43 units in Five university (tertiary) hospitals	1188 nurses	Relationship between staffing, adverse events and missed nursing care	RN4Cast Questionnaire	**Not reported**
Pereira Lima Silva et al. (2020) [59]	Brazil	Cross-sectional	3 large ICUs. 2 from large public institutions and a 3^rd^ from a private hospital offering complex services	29 ICU care nurses	Practice environment and nursing workload	MISSCARE Brasil	**Not reported**
Saqer et al. (2018) (22)	Jordan	Cross-sectional	Six Jordanian hospitals (Government, university, and private hospitals)	362 Hospital nurses	Reasons and predictors of missed care, confidence in delegation	MISSCARE (Arabic-translation)	**Not reported**
Silva et al. (2021) [60]	Brazil	Cross-sectional	Women’s Health Care Unit of a single tertiary (teaching) hospital	62 Nurses	Factors and reasons	MISSCARE-Brasil	**Not reported**
Siqueira et al. (2017) [25]	Brazil	Cross-sectional	Single large-scale tertiary (teaching) hospital	330 nurse aides, technicians, nurses, and nurse administrators	Confirmatory Factor Analysis and factors	MISSCARE Brasil	**Not reported**
Taskiran et al. (2022) [61]	Turkey	Cross-sectional	10 Public, University, and private hospitals	1310 nurses	Frequency, reasons, correlates, and predictors	MISSCARE (Turkish translation)	**2.93 (1.00–4.00)**
Valles et al. (2016) [41]	Mexico	Cross-sectional	A single tertiary hospital	161 Nurses and 483 patients	Factors for missed nursing care	MISSCARE (Adapted for pressure ulcers)	**Not reported**
Zárate-Grajales et al. (2022) [62]	Mexico	Cross-sectional	11 Specialised public hospitals (tertiary) in Mexico	315 nurses	Frequency and factors	MISSCARE	**15.9%**
Zhu et al. (2019) [37]	China	Cross-sectional	Medical and surgical units from 181 hospitals (secondary and tertiary-level)	7802 Nurses	NA	Basel Extent of Rationing of Nursing Care (BERNCA-R)	**3.31 (Not reported)**

Emboldened—Prevalence of missed nursing care presented as mean/median Likert scores are the overall averages of individual nursing task mean Likert scores across a study population of nurses. A scale of 5.00 means a 5-point Likert scale was used, while that of 4.00 means a 4-point Likert Scale was used. Those presented as percentages are either the proportion of nurses who are classified as missing care based on a pre-agreed criteria by individual studies or the proportion of patients who had nursing tasks completed for patient-level estimations

*BERNCA* Basel Extent of Rationing of Nursing Care, *BERNCA-R* Basel Extent of Rationing of Nursing Care—Revised, *MISSCARE* Missed nursing care survey tool, *MNCS* Missed Nursing Care scale

*Indirectly derived, study reported task completion in 14% of babies

### Description of included papers

We included 31 studies in our final analysis; 28 (90.3%) of these were cross-sectional studies, two employed a before and after interventional design, while one was multi-method, employing both cross-sectional and a before and after design (Table 2). Geographically, the greatest number of studies were conducted in Brazil (6 of 31 studies, Table 2). Seven out of the 31 studies were conducted across Africa—Egypt (2), Ethiopia (2), South Africa (1), Nigeria (1) and Kenya (1). Using the World classification for LMIC, 27 out of the 31 studies were from upper-middle income settings, 4 from lower-middle income contexts (Kenya, Egypt, Nigeria) and no study was reported from a low-income country setting (Table 2).

Across all studies, six different missed nursing care tools were used (Table 2). These tools are summarised in Table 1. Twenty-two out of 31 studies (70.9%) used the Missed Nursing Care Survey tool (MISSCARE): 14 in its original form (either in English or translated to a local language)**,** 5 used an adapted Brazilian version, and one each used an adapted Chinese tool, a specifically adapted version to assess maternal health, and one adapted for assessing pressure ulcers (Table 2). Nine other studies each used one of the following tools: Basel Extent of Rationing of Nursing Care (BERNCA-R), Missed Nursing Care Observational Checklist, Nursing Care Index, the RN4Cast Questionnaire, Missed Nursing Care scale (MNCS); one study used an unnamed tool (Table 2). These tools were largely based on nurse or patient self-reports except for 2 studies which used the Missed Nursing Care Observational Checklist and the Nursing Care Index which were both based on direct observations of care provided (Table 1) [27, 36]. Study sample sizes varied considerably and ranged between 28 nurses in one Egyptian study, [36] to 7802 nurses in a Chinese study [37]. Majority of studies were single centre studies and were conducted in tertiary-level hospital settings.

### Quality assessment of included studies

For the selected studies, the quality assessment scores ranged from 2 to 9 out of a maximum score of 10. Based on our classification of high (≥ 7 points), medium (4–6 points) and low (0–3 points) quality studies, 18 (58.1%) of 31 studies were assessed to be high quality, 12 (38.7%) studies assessed as moderate quality and 1 (3.3%) of poor quality (Table 3). The most missed quality assessment criterion was providing information on study non-respondents, 28 of 30 studies did not have any information on this (Table 3). 1 in 2 studies had no information on sample size determination (Table 3).

**Table 3:** Risk of bias assessments using the Newcastle–Ottawa Scale

Study	Selection				Comparability	Outcome		
	Sample representativeness	Sample size	Non-respondents	Exposure (risk factor) ascertainment	Comparable groups. Confounding factors are controlled	Outcome Assessment	Statistical test	Total Score (Maximum – 10)
Al‐Faouri et al. 2021	1	1	0	2	0	1	0	5
Arslan et al. 2021	0	1	0	2	2	1	1	7
Assaye et al., 2022	1	1	0	2	2	1	1	8
Bacaksiz et al. 2020	1	0	0	2	0	1	0	4
Bekker et al., 2015	1	0	0	2	0	1	1	5
Chegini et al. 2020	1	0	0	2	2	1	1	7
Du et al. 2020	1	1	0	2	1	1	1	7
Dutra et al. 2019	1	0	0	2	0	1	0	4
Gathara et al. 2020	1	1	1	1	2	2	1	9
Ghezeljeh et al. 2020	1	1	0	2	2	1	1	8
Haftu et al. 2019	1	1	0	2	2	1	1	8
Hammad et al. 2021	1	1	0	2	0	1	0	5
Hernández-Cruz et al. 2017	1	0	0	2	2	1	1	7
John et al., 2016	1	0	0	0	0	1	0	2
Kalisch et al. 2013	1	0	0	2	2	1	1	7
Kalisch et al. 2020	1	0	1	2	0	1	0	5
Labrague et al. 2021	1	0	0	2	2	1	1	7
Labrague et al., 2022	1	1	0	2	2	1	1	8
Lima et al. 2020	1	0	0	2	0	1	1	5
Moreno-Monsiváis et al. 2015	1	0	0	2	0	1	0	4
Moura et al. 2020	1	0	0	2	0	1	0	4
Nahasaram et al. 2021	1	1	0	2	2	1	1	8
Nantsupawat et al., 2022	1	1	0	2	2	1	1	8
Pereira Lima Silva et al. 2020	1	0	0	2	0	1	0	4
Saqer et al. 2018	1	1	0	2	2	1	1	8
Silva et al. 2021	1	0	0	2	0	1	1	5
Siqueira et al., 2017	1	1	0	2	2	1	1	8
Taskiran et al., 2022	1	1	0	2	2	1	1	8
Valles et al., 2021	1	1	0	2	0	1	1	6
Zárate-Grajales, 2022	0	1	0	2	2	1	1	7
Zhu et al. 2019	1	0	0	2	2	1	1	7

### Prevalence of missed nursing care

Various tools presented varying prevalence of missed nursing care and even when the same tool was employed by different studies, this was derived and reported differently. The MISSCARE tool, for example, asks nurses to rank specific nursing activities missed on either a four-point or 5-point scale (Table 1), where 1 might be a task being rarely missed, up to 5 which means it is always missed. Studies that reported a median/mean Likert score as a proxy for the prevalence of missed nursing care determined an average score for individual nursing activities across a sample population of nurses and determined an overall average across all activities (Table 1) [24, 43]. Those that presented proportions, reported the proportion of nurses who always missed at least one nursing task [47], or the proportion of those who commonly missed care for at least one nursing task (based on dichotomising the Likert scoring into commonly missed and not commonly missed) [40, 62]. Some other studies using other tools, like the Nursing Care Index (NCI), presented patient-level estimates of the proportion of patients who had complete care [27]. Overall, the prevalence of missed nursing care ranged between 15.9 and 86% for studies who reported proportions (Table 2).

### Relative frequency and categories of nursing care missed in LMIC

Seven studies employed the original MISSCARE tool and presented complete data, while six studies did the same for the MISSCARE-Brazil (Table 4). For each nursing task, we compare the within study ranking across the 13 studies and determine an overall median rank (Table 4). The most missed nursing activities based on relative position of the overall median ranks across nursing activities were in the planning and provision of physical needs dimensions of nursing care (Table 4). The 3 least missed nursing care elements were nursing activities classed as assessments (Table 4). Most studies were broadly consistent in the relative rankings of the least and most missed nursing activities except for Chegini et al. [46] an Iranian study conducted across public and private hospital settings. The actual task frequency scores and proportions reported in the original studies are provided along with our nursing task activity rankings in Additional file 3 and Additional file 4.

**Table 4:** Table showing nursing dimensions of care, individual nurse task rank within study, overall rank across primary studies and relative position of activities for studies which used the original MISSCARE survey

Nursing dimensions of care	Nurse activities	Arslan et al.	Nahasaram et al.	Al-Faouri et al.	Hammad et al.	Chegini et al.	Saqer et al.	Kalisch et al.	Lima et al.	Haftu et al.	Lima Silva et al.	Moura et al.	Dutra et al.	Silva et al.	Median rank	Relative position^%^
**Assessments**	**IV/central line site care and assessments**	**24**	**20**	**22**	**24**	**7**	**21**	**21**	**17**	**19**	**15**	**19**	**20**	**19**	**20**	**22nd**
	**Bedside glucose monitoring as ordered**	**17**	**24**	**24**	**21**	**21**	**24**	**23**	**23**	**21**	**24**	**24**	**22**	**23**	**23**	**24th**
	Focused reassessments according to patient condition	5	14	18	8	7	16	19	16	14	13	14	15	19	14	14th
	Monitoring intake/output	18	12	18	20	17	18	10	11	9	21	11	10	6	12	11th
	**Vital signs assessed as ordered**	**23**	**22**	**23**	**23**	**23**	**21**	**24**	**19**	**21**	**21**	**22**	**22**	**24**	**22**	**23rd**
	Patient assessments performed each shift	22	15	18	14	4	18	17	17	11	11	18	12	22	17	18th
	Assess effectiveness of medications	16	10	12	12	21	12	12	9	8	6	16	9	18	12	11th
Emotional support	Emotional support to patient and/or family	8	6	7	11	2	8	3	7	3	5	10	3	16	7	6th
Medical needs	Response to call light is initiated within 5 min	4	17	15	17	4	14	14	6	14	10	4	15	8	14	14th
	PRN medication request acted on within 15 min	14	15	17	15	13	16	15	14	18	4	7	19	14	15	16th
	Wound care	20	23	16	16	19	18	15	24	21	20	13	22	8	19	21st
	Medications administered within 30 min before or after scheduled time	15	13	14	19	7	12	20	13	7	6	12	8	11	12	11th
Physical needs	Setting up meals for patients who can feed themselves	12	10	8	13	23	8	17	10	6	15	3	7	8	10	8th
	**Turning patient every 2 h**	**6**	**4**	**5**	**9**	**7**	**5**	**5**	**4**	**4**	**6**	**5**	**4**	**2**	**5**	**3rd**
	Mouth care	12	5	4	4	7	2	6	8	14	17	6	15	1	6	4th
	Feeding patient when the food is still warm	8	7	3	6	19	2	10	20	11	19	19	12	3	10	8th
	**Ambulation 3 times per day or as ordered**	**1**	**3**	**1**	**2**	**13**	**1**	**2**	**2**	**2**	**1**	**2**	**2**	**7**	**2**	**2nd**
	Assist with toileting needs within 5 min of request	10	8	6	9	7	8	8	15	9	6	8	9	3	8	7th
	Patient bathing/skin care	7	17	11	3	17	8	22	21	19	21	19	20	12	17	18th
**Planning**	**Attending family/interdisciplinary conferences**	**2**	**1**	**2**	**1**	**3**	**2**	**1**	**1**	**1**	**3**	**1**	**1**	**5**	**1**	**1st**
Teaching	Teach patient about plans for their care after discharge and when to call after discharge	3	9	9	7	1	5	13	3	5	2	9	6	13	6	4th
	Patient teaching about procedures, tests, and other diagnostic studies	11	2	10	5	4	7	4	12	14	11	17	15	15	11	10th
Undefined	Hand washing	18	21	13	18	16	14	7	22	21	17	23	22	19	18	20th
	Full documentation of all necessary data	20	19	21	22	13	21	9	5	13	13	15	14	16	15	16th

The individual ranks are missed nursing activities ordered within study; the median rank determines a median across all reported study ranks, while the position compares the relative position of the task based on the calculated median rank

%—Relative positions—1st ranks as the most missed nursing activity, while 24th is the least missed

*Emboldened lines—top and least 3 most missed nursing activities

For studies that used the MNCS (2 studies), the 3 most missed activities were in emotional and physical need categories, while the 3 least missed were all related to provision of medical needs [34, 48]. Other versions of the MISSCARE tool, the MISSCARE modified for pressure ulcers, MISSCARE modified for Obstetrics and Gynaecology, the MISSCARE-Chinese version and the RN4Cast questionnaire, BERNCA, BERNCA-R, the NCI tool were all used by single studies or only had one study report complete data and so were not included in the final synthesis.

### Reasons for missed nursing care in LMIC

Only 6 out of 13 above studies reported on reasons for missed nursing care using the MISSCARE tool (original MISSCARE and MISSCARE Brazil). The most reported reason for missed nursing care across these studies were staffing-related; an inadequate number of nursing staff ranked first, while inadequate number of assistive personnel and unexpected rise in patient volume and/or acuity both ranked 2nd (Additional file 5).

### Factors associated with missed nursing care in LMIC

**Fig. 2:**
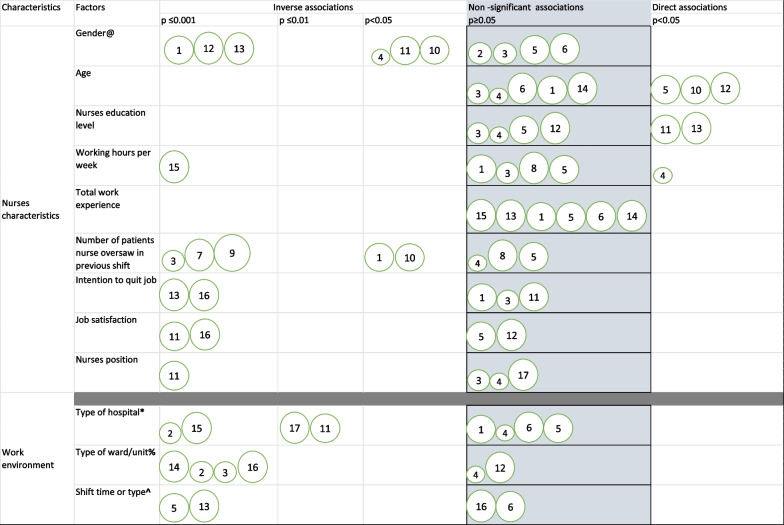
Bubble plot showing factors associated with missed nursing care and the individual studies which reported these factors, their quality (The larger the bubble the higher the study quality), *p* values and direction of association (direct or inverse relationship with missed nursing care). Diagram contains factors that were reported by 4 or more studies. Inverse association means that both the risk factor and missed nursing care go in different directions, for example, higher levels of the factor are associated with less missed nursing care and vice versa. Direct association means both the level of missed nursing care and the factor go in the same direction. @ Gender, all studies report male nurses having greater levels of missed nursing care, except for bubble 11 which reported female nurses as having higher levels. * Type of hospital, greater missed nursing care in public hospitals than private hospitals (Bubble 2 and 15), less in tertiary and specialized care (Bubble 11), less in smaller than larger hospitals (Bubble 17). % Type of ward/unit, greater missed nursing care in surgical than medical wards (Bubble 7), greater levels in general than critical care wards (Bubble 2 and 3), less in closed units—Intensive care, hemato-oncology, bone marrow transplant units (Bubble 16). ^ Later shifts such as night or evening associated with greater missed nursing care than day shifts

Multiple factors were studied to identify their associations with missed nursing care. We grouped this based on nurse and workplace characteristics. The most studied factor was nurses’ gender (Fig. 2), and this was significant in 6 out of 10 studies which suggested male nurses were more likely to miss patient care (Fig. 2) [22, 26, 40, 43, 46, 44]. Similarly, the number of patients the nurse oversaw in their last shift was a commonly investigated risk factor and a higher order of patients was associated with greater missed nursing care in 5 out of 8 studies [27, 43, 46, 49, 58]. Other nursing characteristics such as nurses age, educational level and total work experience were not significantly associated with missed nursing care when examined (Fig. 2). Type of hospital and unit/ward were the most studied work environment characteristics and demonstrated mixed associations with missed nursing care (Fig. 2). Overall quality of the studies did not affect whether factors were significantly associated with missed nursing care.

## Discussion

Our systematic review identified 31 papers that described missed nursing care in LMIC acute hospital settings. Majority of these studies were from tertiary care contexts and were cross-sectional. These studies were also from upper middle-income country settings. There were only 2 interventional studies of low to moderate quality [36, 55]. Studies were also largely conducted in adult surgical and medical units or in Intensive Care Units (ICUs), with limited data from other care settings. This perhaps relates to measurement tools for missed nursing care being developed in adult care settings. We noted a few modifications by some studies to measure the concept in alternate care settings, for example, the MISSCARE tool was modified for use in obstetrics [40], and one study developed a tool specifically for missed care in newborn settings [27]. This highlights a need for tools that can be employed across multiple care settings to provide a more complete understanding of this phenomenon.

The prevalence of missed nursing care varied from 15.2 to 86.0%. It was, however, difficult to make meaningful comparisons across studies or compare our findings with data from high-income countries. This was in part due to a lack of consistency in how missed care was measured, defined, and reported across the reviewed studies. This non-uniformity is not unique to LMIC but is ubiquitous across the missed nursing care research landscape [63]. This review identified six different measurement tools which differed in the specific nursing activities they measured. Even when studies employed the same tools, their definitions and reporting of missed nursing care differed. For example, studies that used the MISSCARE tool reported a median or mean Likert score based on nurses self-report of care they missed in previous shifts [24, 26, 57], or dichotomized scores to determine a proportion of nurses who missed care [46, 55, 59]. Some other prevalence estimates were derived at patient-level and not on nurse-self report [27].

Another challenge was many studies had small sample sizes and used tools that were based on nurses’ self-reporting of care they missed during their previous shifts. Recall and social desirability bias are known challenges associated with self-reported outcome assessments. In some high-income settings, these self-reported tools have been used in multi-center observational studies, where validity arguments are strengthened by demonstrating high intraclass correlation coefficients within units of analysis, such as, for example, nurses who work in the same wards having similar missed nursing care experiences [39]. Validation studies have also shown evidence of good predictive ability of self-reported missed nursing care tools, suggesting nurses provide accurate and reliable information on nurse staffing, missed care and experience of adverse events using self-report surveys [25, 64]. We found only 2 studies employed tools which were based on direct observations of care, the Nursing Care Index, and the Missed Nursing Care Observation tool [27, 36]. Although, these potentially provide a more accurate reflection of missed nursing care particularly with smaller sized studies, they are comparatively difficult to undertake when compared to administering questionnaires (which are the basis for the nurse self-report data) and investigators would need to manage the Hawthorne effect, a direct consequence of observation [65].

To mitigate the challenges encountered with direct comparisons across studies, we rank ordered the activities missed within studies using the same tool and calculated a median rank across studies with complete data. As such, we were able to summarise the findings form a subset of studies that used either the MISSCARE or MISSCARE-Brasil tool. This subset was similar to the underlying data as they mainly came from tertiary care settings and adult medical and surgical settings but had a higher proportion of studies conducted in Brazil. Grouping nursing activities within these tools using the American Nurses Association classification for nursing activities allowed us to identify broad dimensions of least and most missed nursing activities. We noted the least missed care activities were clinical nursing assessments and the most missed were planning; specifically attending interdisciplinary patient conferences and providing for patient physical needs. This is similar to the finding from reviews reporting data from high-income countries [4, 13]. This suggests patterns of care prioritisation related to missed nursing care are broadly similar across diverse contexts and perhaps related to the training or socialization of nurses. Such clinical prioritization, however, undermines provision of holistic nursing care.

From a policy perspective, our finding showing attention to patient physical needs as one of the most missed nursing care activities might suggest a space for formal task shifting for these low priority potentially lower skill activities. Ethnographic work from some LMIC settings suggest that low priority nursing activities are already being informally transferred to unqualified persons, such as patient relatives, hospital support staff and students without structured supervision [66]. In theory, increasing support staffing could provide nurses the extra time they need to focus on high priority nursing activities. The counter argument to this is nursing activities viewed as low priority, for example, patient comfort, feeding and elimination care are central tenets of nursing practice and components of fundamental nursing care [67]. Although there is some data from high-income countries to support task shifting [68], contextual research conducted in LMIC will be needed to explore such arguments. Forms of task-shifting may need to be regulated to avoid blurring of roles and supportive staff would need to be under the direct supervision of nurses, to ensure patient safety. Some physical nursing tasks, for example, turning of patients regularly to prevent pressure injuries and prevention of falls rely on a skilled situational assessment by clinically trained nurses. In these instances, support staff might act to implement the nurses’ orders.

We noted a smaller subset of studies (n = 6) which reported on the reasons for missed nursing care as put forward by nurses using the MISSCARE tool. These were all labour-related reasons and included inadequate numbers of nurses and nurse assistive personnel and an unexpected rise in patient care numbers. Although the smaller numbers limit generalizability, they speak to the importance of poor staff to patient ratios in many LMIC; in the more resource constrained settings ratios have been reported to be as extreme as 1 nurse caring for around 25 patients [27, 69]. Studies that investigate the role nurse staffing plays in missed nursing care within these environments would be helpful.

Although clinical assessments were the least missed in relative terms. The individual data from studies show even high priority activities, such as patient monitoring are missed, and this might have the greatest threat to patient safety. For example, although in one study, patient assessments were the least missed they were still reportedly missed by 16% of nurses [44].

The most widely reported nurse-level factors associated with missed care were age, gender, education level, working hours per week, nurses’ work experience, intention to quit job and number of patients the nurse cared for in their previous shift. Overall, studies largely reported non-significant associations with nurse-level characteristics and missed nursing care except for two characteristics—gender (male nurses miss more care, Refer to Fig. 2) and number of patients cared for in the previous shift [43, 46, 49, 58]. Type of hospital, ward or unit and the nursing shift time or type were the most frequently explored work environment factors, and this showed a largely mixed picture. Studies that reported significant relationship with missed nursing care showed it was more prevalent in government-owned (public) hospitals [23], while tertiary specialist hospitals had comparatively less missed nursing care compared to other hospital types [26]. Similarly, missed nursing care was less in intensive care wards than regular wards and greater on night and evening nursing shifts than the day shift [24, 40, 62].

The literature on missed nursing care in LMIC in this review comprise observational studies that describe the existing problem. Only two studies focused on interventions to improve missed care and both of these scored low on our risk of bias scores [42, 55]. Paucity of intervention research to address missed nursing care is not unique to LMIC but has been reported globally [70]. One recent review on interventions for missed nursing care reported only 13 studies, all from high-income countries settings. [11]. There is currently some ongoing prospective interventional research to investigate if increasing the number of nurses in a resource constrained LMIC setting might reduce missed nursing care [71].

### Strengths and limitation

To the best of the authors knowledge, this is the first review to integrate knowledge on missed nursing care in LMIC settings. We note that the data that we present came from mainly upper middle-income settings, and we are unable to make conclusions for low and lower-middle income settings due to limited data from these settings. Our review was also limited to English due to translation limitations on the team. In addition, due to the multiple forms of missed nursing care tools employed which differed in length, questions they assessed and completeness, we were only able to pool together a fraction of studies to determine the most missed nursing care categories.

## Conclusions

There is a lack of standardization in the measurement of missed nursing care in LMIC and the current tools are not transferrable across care settings. The existing data are mainly from upper-middle income country settings and most existing tools are based on nurses self-reporting.

We found clinical nursing activities to be the least missed, while non-clinical patient needs were most missed. This undermines the concept of holistic nursing but also suggests a possible space for carefully designed task-shifting. There is a need for contextual research in LMIC to determine the effects of increasing nurse numbers or adding nurse support workers might have on missed nursing care. To allow for a greater universal understanding of the concept, specific research needs to be conducted in low-income country settings.

## Supplementary Information

**Additional file 1.** Systematic review search strategy.

**Additional file 2.** List of papers excluded and reasons for their exclusion.

**Additional file 3.** Relative frequency of missed nursing activities and ranking of studies employing the original MISSCARE tool.

**Additional file 4.** Relative frequency of missed nursing activities and ranking of studies employing the MISSCARE Brazil tool.

**Additional file 5.** Table showing ranked nurse self-reported reasons for missed nursing care using the MISSCARE instrument, median rank across primary studies and relative position of reasons for missed nursing care. (The median rank is the median of all individual study ranks across all studies, while the position compares the relative position of each reason based on the median rank).

**Additional file 6.** PRISMA Checklist.

## Data Availability

This study is developed from publicly available secondary data and no primary data were generated or analysed for this study. All relevant data for this study are either included in the figures and tables or have been uploaded as online Additional information.

## Abbreviations

BERNCA-R: Basel Extent of Rationing of Nursing Care Revised
CINAHL: Cumulative Index to Nursing and Allied Health Literature
ICUs: Intensive Care Units
LMIC: Low-income and middle-income countries
MISSCARE: Missed nursing care survey
MNCS: Missed Nursing Care scale
NCI: Nursing Care Index
PROSPERO: International Prospective Register of Systematic Reviews
WHO: World Health Organization
