# Supplementary material for: Missed nursing care in acute care hospital settings in low-income and middle-income countries: a systematic review
Source: Hum Resour Health. 2023 Mar 14;21:19. doi: 10.1186/s12960-023-00807-7 (PMC10015781; doi:10.1186/s12960-023-00807-7)
Supplement: Supplementary file 1 — Additional file 1. Systematic review search strategy. [file 12960_2023_807_MOESM1_ESM.pdf]

S1 Table - Systematic review search strategy

Medline and Embase search strategy for missed nursing care in Acute care hospital settings in low-middle income country settings

1. exp DEVELOPING COUNTRIES/
2. "developing countr* ".ab,kw,ti.
3. "developing world* ".ab,kw,ti.
4. "LMIC*".ab,kw,ti.
5. "low* income* ".ab,kw,ti.
6. "middle income".ab,kw,ti.
7. "resource poor".ab,kw,ti.
8. "resource limited".ab,kw,ti.
9. exp AFRICA/
10. (Africa or Algeria* or Angola* or Benin or Botswana* or Burkina or Burundi* or "Cabo Verde" or "Cape Verde" or Cameroon* or "Central African Republic" or Chad or Comoros or Congo* or "Cote d'Ivoire" or Djibouti or Egypt* or Guinea* or Eritrea* or Eswatini or Swaziland or Ethiopia* or Gabon or Gambia* or Ghana* or Kenya* or Lesotho or Liberia* or Libya* or Madagascar or Malawi* or Mali or Mauritania* or Mauritius or Morocc* or Mozambique or Namibia* or Niger or Nigeria* or Rwanda* or "Sao Tome and Principe" or Senegal* or Seychelles or "Sierra Leone" or Somalia or Sudan* or Tanzania* or Togo or Tunisia* or Uganda* or Zambia* or Zimbabwe* or Southafrica* or Maghreb* or Sahara* or SubSahara*).ab,in,kw,ti.
11. exp ASIA/
12. exp FAR EAST/
13. 13 exp ASIA, CENTRAL/
14. exp ASIA, SOUTH/
15. exp ASIA, SOUTHEASTERN/
16. exp ASIA, WESTERN/
17. exp MIDDLE EAST/
18. exp TRANSCAUCASIA/

19. (Afghan* or Armenia* or Azerbaijan* or Bangladesh* or Bhutan* or Cambodia* or China or Chinese or "North Korea*" or "People* Republic of Korea" or Georgia* or India or Indian or Indonesia* or Iran* or Iraq* or Jordan* or Kazakhstan* or Kyrgyz* or Laos or Lao or Leban* or Malay* or Maldives or Mongolia* or Myanmar or Burm* or Nepal* or Pakistan* or Philippines or "Sri Lanka*" or Syria* or Tajik* or Thailand or Thai or Turkey or Turkish or Turkmen* or Uzbek* or Vietnam* or Gaza or "West Bank" or Palestin* or Yemen*).ab,in,kw,ti
20. exp LATIN AMERICA/
21. exp SOUTH AMERICA/
22. exp CENTRAL AMERICA/
23. exp CARIBBEAN REGION/
24. MEXICO/
25. (Argentin* or Belize or Bolivia* or Brazil* or Colombia* or Cuba* or Chile* or "Costa Rica*" or Dominica* or Ecuador* or Salvador* or Grenada* or Guatemala* or Guyana* or Haiti* or Hondura* or Jamaica* or Mexico or Mexican* or Nicaragua* or Paraguay* or Peru or Peruvian* or "St* Lucia*" or "St* Vincent" or Suriname or Uruguay* or Venezuela*).ab,in,kw,ti.
26. exp PACIFIC ISLANDS/
27. exp POLYNESIA/
28. exp MELANESIA/
29. exp MICRONESIA/
30. (Samoa* or Fiji* or Kiribati* or "Marshall Island*" or Micronesia* or Nauru* or "Papua New" or Polynesia* or "Solomon Island*" or "Timor Leste" or Tonga* or Tuvalu* or Vanuatu*).ab,in,kw,ti.
31. exp RUSSIA/
32. ALBANIA/ or "BOSNIA AND HERZEGOVINA"/ or BELARUS/ or BULGARIA/ or KOSOVO/ or MOLDOVA/ or MONTENEGRO/ or "REPUBLIC OF NORTH MACEDONIA"/ or ROMANIA/ or SERBIA/ or UKRAINE/
33. (Russia* or Albania* or Bosnia* or Belarus* or Bulgaria* or Kosovo or Kosova* or Moldova* or Montenegr* or "North Macedonia*" or Romania* or Serbia* or Ukrain*).ab,in,kw,ti.
34. 1 or 2 or 3 or 4 or 5 or 6 or 7 or 8 or 9 or 10 or 11 or 12 or 13 or 14 or 15 or 16 or 17 or 18 or 19 or 20 or 21 or 22 or 23 or 24 or 25 or 26 or 27 or 28 or 29 or 30 or 31 or 32 or 33

35.	JAPAN/ or SINGAPORE/ or TAIWAN/ or REPUBLIC OF KOREA/ or BAHRAIN/ or ISRAEL/ or KUWAIT/ or QATAR/ or SAUDI ARABIA/ or UNITED ARAB EMIRATES/
36.	AUSTRALIA/ or SOUTH AUSTRALIA/ or WESTERN AUSTRALIA/ or NEW ZEALAND/ or HAWAII/ or NEW CALEDONIA/
37.	35 or 36
38.	38 34 not 37
39.	39 (nurse* or nursing).ti.
40.	38 and 39
41.	41 (missed or missing).ti.
42.	(lack or lacking).ti.
43.	(shortage or absence).ti.
44.	(unmet or incomplete).ti.
45.	"delay*".ti.
46.	(staffing or error* or fail* or omission* or rationing or unfulfil*).ti.
47.	41 or 42 or 43 or 44 or 45 or 46
48.	40 and 47
49.	limit 48 to yr="2011 -Current"
50.	limit 49 to conference abstracts
51.	49 not 50

CINAHL search strategy for missed nursing care in Acute care hospital settings in low-middle income country settings

#	Query
S6	S3 AND S4 AND S5
S5	S1 OR S2
S4	TI (missed or missing) OR TI (lack or lacking) OR TI (staffing or error* or fail* or omission* or rationing or unfulfil*) TI (unmet or incomplete) OR TI (shortage or absence) OR TI (delay*) OR TI (medical error*) OR AB (medical error*) OR KW (medical error*) OR MH "Health Care Rationing"/
S3	TI (nurse* or nursing) OR MH "Nursing care+"/ OR MH "Nursing Staff, Hospital/" OR MH "Personnel Staffing and Scheduling+"/
S2	(MH "AFRICA+"/ OR TI (Africa or Algeria* or Angola* or Benin or Botswana* or Burkina or Burundi* or "Cabo Verde" or "Cape Verde" or Cameroon* or "Central African Republic" or Chad or Comoros or Congo* or "Cote d'Ivoire" or Djibouti or Egypt* or Guinea* or Eritrea* or Eswatini or Swaziland or Ethiopia* or Gabon or Gambia* or Ghana* or Kenya* or Lesotho or Liberia* or Libya* or Madagascar or Malawi* or Mali or Mauritania* or Mauritius or Morocc* or Mozambique or Namibia* or Niger or Nigeria* or Rwanda* or "Sao Tome and Principe" or Senegal* or Seychelles or "Sierra Leone" or Somalia or Sudan* or Tanzania* or Togo or Tunisia* or Uganda* or Zambia* or Zimbabwe* or Southafrica* or Maghreb* or Sahara* or SubSahara*) OR MH "ASIA+"/ OR MH "FAR EAST+"/ OR MH "ASIA, CENTRAL+"/ OR MH "ASIA, SOUTH+"/ OR MH "ASIA, SOUTHEASTERN+"/ OR MH "ASIA, WESTERN+"/ OR MH "MIDDLE EAST+"/ OR MH "TRANSCAUCASIA+"/ OR TI (Afghan* or Armenia* or Azerbaijan* or Bangladesh* or Bhutan* or Cambodia* or China or Chinese or "North Korea*" or "People* Republic of Korea" or Georgia* or India or Indian or Indonesia* or Iran* or Iraq* or Jordan* or Kazakhstan* or Kyrgyz* or Laos or Lao or Leban* or Malay* or Maldives or Mongolia* or Myanmar or Burm* or Nepal* or Pakistan* or Philippines or "Sri Lanka*" or Syria* or Tajik* or Thailand or Thai or Turkey or Turkish or Turkmen* or Uzbek* or Vietnam* or Gaza or "West Bank" or Palestin* or Yemen*)) OR (MH "LATIN AMERICA+"/ OR MH "SOUTH AMERICA+"/ OR MH "CENTRAL AMERICA+"/ OR MH "CARIBBEAN REGION+"/ OR MH "MEXICO"/ OR TI (Argentin* or Belize or Bolivia* or Brazil* or Colombia* or Cuba* or Chile* or "Costa Rica*" or Dominica* or Ecuador* or Salvador* or Grenada* or Guatemala* or Guyana* or Haiti* or Hondura* or

	Jamaica* or Mexico or Mexican* or Nicaragua* or Paraguay* or Peru or Peruvian* or "St* Lucia*" or "St* Vincent" or Suriname or Uruguay* or Venezuela*) OR MH "PACIFIC ISLANDS+"/ OR MH "POLYNESIA+"/ OR MH "MELANESIA+"/ OR MH "MICRONESIA+"/ OR TI (Samoa* or Fiji* or Kiribati* or "Marshall Island*" or Micronesia* or Nauru* or "Papua New" or Polynesia* or "Solomon Island*" or "Timor Leste" or Tonga* or Tuvalu* or Vanuatu*) OR MH "RUSSIA+"/ OR MH (ALBANIA/ or "BOSNIA AND HERZEGOVINA"/ or BELARUS/ or BULGARIA/ or KOSOVO/ or MOLDOVA/ or MONTENEGRO/ or "REPUBLIC OF NORTH MACEDONIA"/ or ROMANIA/ or SERBIA/ or UKRAINE/) OR TI (Russia* or Albania* or Bosnia* or Belarus* or Bulgaria* or Kosovo or Kosova* or Moldova* or Montenegr* or "North Macedonia*" or Romania* or Serbia* or Ukrain*)) OR (AB (Africa or Algeria* or Angola* or Benin or Botswana* or Burkina or Burundi* or "Cabo Verde" or "Cape Verde" or Cameroon* or "Central African Republic" or Chad or Comoros or Congo* or "Cote d'Ivoire" or Djibouti or Egypt* or Guinea* or Eritrea* or Eswatini or Swaziland or Ethiopia* or Gabon or Gambia* or Ghana* or Kenya* or Lesotho or Liberia* or Libya* or Madagascar or Malawi* or Mali or Mauritania* or Mauritius or Morocc* or Mozambique or Namibia* or Niger or Nigeria* or Rwanda* or "Sao Tome and Principe" or Senegal* or Seychelles or "Sierra Leone" or Somalia or Sudan* or Tanzania* or Togo or Tunisia* or Uganda* or Zambia* or Zimbabwe* or Southafrica* or Maghreb* or Sahara* or SubSahara*) OR AB (Afghan* or Armenia* or Azerbaijan* or Bangladesh* or Bhutan* or Cambodia* or China or Chinese or "North Korea*" or "People* Republic of Korea" or Georgia* or India or Indian or Indonesia* or Iran* or Iraq* or Jordan* or Kazakhstan* or Kyrgyz* or Laos or Lao or Leban* or Malay* or Maldives or Mongolia* or Myanmar or Burm* or Nepal* or Pakistan* or Philippines or "Sri Lanka*" or Syria* or Tajik* or Thailand or Thai or Turkey or Turkish or Turkmen* or Uzbek* or Vietnam* or Gaza or "West Bank" or Palestin* or Yemen*) OR AB (Argentin* or Belize or Bolivia* or Brazil* or Colombia* or Cuba* or Chile* or "Costa Rica*" or Dominica* or Ecuador* or Salvador* or Grenada* or Guatemala* or Guyana* or Haiti* or Hondura* or Jamaica* or Mexico or Mexican* or Nicaragua* or Paraguay* or Peru or Peruvian* or "St* Lucia*" or "St* Vincent" or Suriname or Uruguay* or Venezuela*) OR AB (Samoa* or Fiji* or Kiribati* or "Marshall Island*" or Micronesia* or Nauru* or "Papua New" or Polynesia* or "Solomon Island*" or "Timor Leste" or Tonga* or Tuvalu* or Vanuatu*) OR AB (Russia* or Albania* or Bosnia* or Belarus* or Bulgaria* or Kosovo or Kosova* or Moldova* or Montenegr* or "North Macedonia*" or Romania* or Serbia* or Ukrain*) OR KW (Africa or Algeria* or Angola* or Benin or Botswana* or Burkina or Burundi* or "Cabo Verde" or "Cape Verde" or Cameroon* or "Central African Republic" or Chad or Comoros or Congo* or "Cote d'Ivoire" or Djibouti or Egypt* or Guinea* or Eritrea* or Eswatini or Swaziland or Ethiopia* or Gabon or Gambia* or Ghana* or Kenya* or Lesotho or Liberia* or Libya* or Madagascar or Malawi* or Mali or Mauritania* or Mauritius or Morocc* or Mozambique or Namibia* or Niger or Nigeria* or Rwanda* or "Sao Tome and Principe" or Senegal* or Seychelles or "Sierra Leone" or Somalia or Sudan* or Tanzania* or Togo or Tunisia* or Uganda* or Zambia* or Zimbabwe* or Southafrica* or Maghreb* or Sahara* or SubSahara*) OR KW (Afghan* or Armenia* or Azerbaijan* or Bangladesh* or Bhutan* or Cambodia* or China or Chinese or "North Korea*" or "People* Republic of Korea" or Georgia* or India or Indian or

	Indonesia* or Iran* or Iraq* or Jordan* or Kazakhstan* or Kyrgyz* or Laos or Lao or Leban* or Malay* or Maldives or Mongolia* or Myanmar or Burm* or Nepal* or Pakistan* or Philippines or "Sri Lanka*" or Syria* or Tajik* or Thailand or Thai or Turkey or Turkish or Turkmen* or Uzbek* or Vietnam* or Gaza or "West Bank" or Palestin* or Yemen*) OR KW (Argentin* or Belize or Bolivia* or Brazil* or Colombia* or Cuba* or Chile* or "Costa Rica*" or Dominica* or Ecuador* or Salvador* or Grenada* or Guatemala* or Guyana* or Haiti* or Hondura* or Jamaica* or Mexico or Mexican* or Nicaragua* or Paraguay* or Peru or Peruvian* or "St* Lucia*" or "St* Vincent" or Suriname or Uruguay* or Venezuela*) OR KW (Samoa* or Fiji* or Kiribati* or "Marshall Island*" or Micronesia* or Nauru* or "Papua New" or Polynesia* or "Solomon Island*" or "Timor Leste" or Tonga* or Tuvalu* or Vanuatu*) OR KW (Russia* or Albania* or Bosnia* or Belarus* or Bulgaria* or Kosovo or Kosova* or Moldova* or Montenegr* or "North Macedonia*" or Romania* or Serbia* or Ukrain*)) NOT (MH ("JAPAN/ or SINGAPORE/ or TAIWAN/ or REPUBLIC OF KOREA/ or BAHRAIN/ or ISRAEL/ or KUWAIT/ or QATAR/ or SAUDI ARABIA/ or UNITED ARAB EMIRATES/ or AUSTRALIA/ or SOUTH AUSTRALIA/ or WESTERN AUSTRALIA/ or NEW ZEALAND/ or HAWAII/ or NEW CALEDONIA/))
S1	(MH "DEVELOPING COUNTRIES+"/ OR TI ("developing countr* ") OR TI ("developing world* ") OR TI ("LMIC*") OR TI ("low* income* ") OR TI ("middle income") OR TI ("resource poor") OR TI ("resource limited") OR TI ("resource constrained")) OR (AB ("developing countr* ") OR AB ("developing world* ") OR AB ("LMIC*") OR AB ("low* income* ") OR AB ("middle income") OR AB ("resource poor") OR AB ("resource limited") OR AB ("resource constrained")) OR (KW ("developing countr* ") OR KW ("developing world* ") OR KW ("LMIC*") OR KW ("low* income* ") OR KW ("middle income") OR KW ("resource poor") OR KW ("resource limited") OR KW ("resource constrained"))
