# Supplementary material for: Missed nursing care in acute care hospital settings in low-income and middle-income countries: a systematic review
Source: Hum Resour Health. 2023 Mar 14;21:19. doi: 10.1186/s12960-023-00807-7 (PMC10015781; doi:10.1186/s12960-023-00807-7)
Supplement: Supplementary file 2 — Additional file 2. List of papers excluded and reasons for their exclusion. [file 12960_2023_807_MOESM2_ESM.pdf]

S2 Table: List of papers excluded and reasons for their exclusion

S/N	Citation	Reason for exclusion
1	Cho SH, Kim YS, Yeon KN, You SJ, Lee ID. Effects of increasing nurse staffing on missed nursing care. International nursing review. 2015 Jun;62(2):267-74.	High income setting (south Korea)
2	Lake ET, French R, O'Rourke K, Sanders J, Srinivas SK. Linking the work environment to missed nursing care in labour and delivery. Journal of nursing management. 2020 Nov;28(8):1901-8.	HIC Setting (US)
3	Khajooee R, Bagherian B, Dehghan M, AZIZZADEH FM. Missed nursing care and its related factors from the points of view of nurses affiliated to Kerman University of Medical Sciences in 2017.	Arabic (non-English)
4	Yaghoubi M, Torki ME, Salesi M, Ehsani-Chimeh E, Bahadori M. the relationship between teamwork and missed nursing care: Case study in a military hospital in Tehran. Journal of Military Medicine. 2019;21(1):63-72.	Arabic (non-English)
5	Blizzard HL. The relationship between the perceptions of implicit rationing of nursing care and emotionally intelligent leadership style among direct-care nurses (Doctoral dissertation).	HIC Setting (US)
6	Tou YH, Liu MF, Chen SR, Lee PH, Kuo LM, Lin PC. Investigating missed care by nursing aides in Taiwanese long-term care facilities. Journal of nursing management. 2020 Nov;28(8):1918-28.	Focused on nursing homes and not acute care settings
7	Siqueira LD, Caliri MH, Kalisch B, Dantas RA. Cultural adaptation and internal consistency analysis of the MISSCARE Survey for use in Brazil1. Revista latino-americana de enfermagem. 2013 Mar;21:610-7.	Portuguese (non-English)
8	Dutra CK, Guirardello ED. Nurse work environment and its impact on reasons for missed care, safety climate, and job satisfaction: A cross-sectional study. Journal of Advanced Nursing. 2021 May;77(5):2398-406.	Focus is on nurse work environment
9	Zhi LI, Chen H, Deai YU. Status quo of nursing lack in level three first-class hospitals of Zhongshan city. Chinese Journal of Practical Nursing. 2013 Jan 1;29(23):51-3.	Chinese (non-english)
10	You L, Zheng J, Liu K, Liu J, Wang Y, Lin X, Zheng L, Pei D. The prevalence and change of rationing of nursing care in level 2 and level 3 hospitals across Guangdong province. Chinese Journal of Practical Nursing. 2016:1166-9.	Chinese (non-english)
11	Kalánková D, Suhonen R, Stolt M, Kurucová R, Katajisto J, Žiaková K, Gurková E. Psychometric testing of perceived implicit rationing of nursing care (PIRNCA). Journal of advanced nursing. 2020 Jun;76(6):1469-82.	HIC setting (Slovak republic)
