# Supplementary material for: Missed nursing care in acute care hospital settings in low-income and middle-income countries: a systematic review
Source: Hum Resour Health. 2023 Mar 14;21:19. doi: 10.1186/s12960-023-00807-7 (PMC10015781; doi:10.1186/s12960-023-00807-7)
Supplement: Supplementary file 3 — Additional file 3. Relative frequency of missed nursing activities and ranking of studies employing the original MISSCARE tool. [file 12960_2023_807_MOESM3_ESM.pdf]

S3 Table - Relative frequency of missed nursing tasks and ranking of studies employing the original MISSACRE tool

Nurse tasks	Arslan et al (2021)	**Arslan Rank**	Nahasaram et al	Nahasaram Rank	Al-Faouri et al	**Al-Faouri Rank**	Hammad et al	**Hammad rank**	Chegini et al	**Chegini rank**	Saqeer et al	**Saqeer rank**	Kalisch et al	**Kalisch rank**
Assess effectiveness of medication	1.14	**16**	1.83	**10**	1.99	**12**	2.3	**12**	1.8	**21**	2.8	**12**	1.24	**12**
Turning patient every 2 h	1.61	**6**	2.36	**4**	2.6	**5**	2.44	**9**	2.9	**7**	3.1	**5**	1.52	**5**
Mouth care	1.22	**12**	2.31	**5**	2.86	**4**	2.64	**4**	2.9	**7**	3.2	**2**	1.46	**6**
PRN medication requests acted on within 15 min	1.2	**14**	1.78	**15**	1.82	**17**	2.06	**15**	2.8	**13**	2.6	**16**	1.18	**15**
IV/central line site care and assessments according to hospital policy	1.06	**24**	1.66	**20**	1.63	**22**	1.7	**24**	2.9	**7**	2.4	**21**	1.12	**21**
Medications administered within 30 min before or after scheduled time	1.15	**15**	1.8	**13**	1.96	**14**	1.96	**19**	2.9	**7**	2.8	**12**	1.13	**20**
Patient bathing/skin care	1.46	**7**	1.74	**17**	2.25	**11**	2.65	**3**	2.1	**17**	2.9	**8**	1.09	**22**

Monitoring intake/output	1.12	**18**	1.82	**12**	1.67	**18**	1.9	**20**	2.1	**17**	2.5	**18**	1.3	**10**
Vital signs assessed as ordered	1.08	**23**	1.45	**22**	1.52	**23**	1.74	**23**	1.7	**23**	2.4	**21**	1.06	**24**
Hand washing	1.12	**18**	1.53	**21**	1.98	**13**	1.99	**18**	2.2	**16**	2.7	**14**	1.4	**7**
Patient assessments performed each shift	1.09	**22**	1.78	**15**	1.67	**18**	2.26	**14**	3	**4**	2.5	**18**	1.16	**17**
Wound care	1.11	**20**	1.42	**23**	1.87	**16**	2.01	**16**	1.9	**19**	2.5	**18**	1.18	**15**
Bedside glucose monitoring as ordered	1.13	**17**	1.34	**24**	1.47	**24**	1.88	**21**	1.8	**21**	2.3	**24**	1.07	**23**
Focused reassessments according to patient condition	1.63	**5**	1.79	**14**	1.67	**18**	2.47	**8**	2.9	**7**	2.6	**16**	1.14	**19**
Patient teaching about procedures, tests, and other diagnostic studies	1.29	**11**	2.44	**2**	2.29	**10**	2.59	**5**	3	**4**	3	**7**	1.58	**4**
Emotional support to patient and/or family	1.45	**8**	2.19	**6**	2.36	**7**	2.41	**11**	3.2	**2**	2.9	**8**	1.63	**3**

Teach patient about plans for their care after discharge and when to call after discharge	1.93	**3**	1.91	**9**	2.31	**9**	2.5	**7**	3.3	**1**	3.1	**5**	1.23	**13**
Attending family conferences/ interdisciplinary conferences	2.26	**2**	2.46	**1**	3.19	**2**	2.84	**1**	3.1	**3**	3.2	**2**	1.85	**1**
Feeding patient when the food is still warm	1.45	**8**	2.1	**7**	2.98	**3**	2.58	**6**	1.9	**19**	3.2	**2**	1.3	**10**
Ambulation 3 times per day or as ordered	2.35	**1**	2.38	**3**	3.26	**1**	2.72	**2**	2.8	**13**	3.3	**1**	1.82	**2**
Assist with toileting needs within 5 min of request	1.37	**10**	1.94	**8**	2.59	**6**	2.44	**9**	2.9	**7**	2.9	**8**	1.32	**8**
Response to call light is initiated within 5 min	1.81	**4**	1.74	**17**	1.91	**15**	2	**17**	3	**4**	2.7	**14**	1.19	**14**
Setting up meals for patients who can feed themselves	1.22	**12**	1.83	**10**	2.33	**8**	2.29	**13**	1.7	**23**	2.9	**8**	1.16	**17**
Full documentation	1.11	**20**	1.73	**19**	1.66	**21**	1.76	**22**	2.8	**13**	2.4	**21**	1.31	**9**

of all necessary data														

Emboldened – individual study rank ordering
