# Supplementary material for: Missed nursing care in acute care hospital settings in low-income and middle-income countries: a systematic review
Source: Hum Resour Health. 2023 Mar 14;21:19. doi: 10.1186/s12960-023-00807-7 (PMC10015781; doi:10.1186/s12960-023-00807-7)
Supplement: Supplementary file 4 — Additional file 4. Relative frequency of missed nursing activities and ranking of studies employing the MISSCARE Brazil tool. [file 12960_2023_807_MOESM4_ESM.pdf]

S4 Table- Relative frequency of missed nursing tasks and ranking of studies employing the MISSACRE Brazil tool

Nurse tasks	Lima et al	**Lima ranking**	Haftu et al	**Haftu ranking**	Lima Silva et al	**Lima Silva ranking**	Moura et al	**Moura rank**	Dutra et al (2019)	**Dutra rank**	Silva et al	**Silva rank**
Assess effectiveness of medications	32.9	**10**	8.67	**9**	31	**7**	14.6	**20**	8.62	**10**	85.5	**22**
Turning patient every 2 h	43.6	**5**	24.14	**4**	31	**7**	39.6	**7**	24.14	**4**	48.4	**3**
Mouth care	34.6	**9**	5.17	**15**	14	**19**	37.5	**8**	5.17	**15**	42	**1**
PRN medication requests acted on within 15 min	21.8	**15**	3.45	**22**	38	**4**	34.4	**9**	3.45	**19**	77.4	**18**
IV/central line site care and assessments according to hospital policy	19	**18**	1.72	**19**	17	**17**	9.4	**23**	1.72	**20**	88.8	**23**
Medications administered within 30 min before or after scheduled time	22.8	**14**	12.07	**8**	31	**7**	22.9	**16**	12.07	**8**	74.1	**14**
Patient bathing/skin care	6.4	**25**	1.72	**19**	3	**25**	9.4	**23**	1.72	**20**	74.2	**15**

Monitoring intake/output	28.2	**12**	8.62	**10**	3	**25**	25	**15**	8.62	**10**	64.5	**7**
Vital signs assessed as ordered	13.9	**21**	0	**22**	3	**25**	5.2	**26**	0	**22**	98.4	**28**
Hand washing	6.3	**26**	0	**22**	14	**19**	4.2	**27**	0	**22**	88.8	**23**
Patient assessments performed each shift	19	**18**	6.9	**12**	24	**12**	10.4	**22**	6.9	**12**	90.3	**26**
Wound care	1.3	**28**	0	**22**	7	**24**	21.9	**17**	0	**22**	70.9	**10**
Bedside glucose monitoring as ordered	3.8	**27**	0	**22**	0	**28**	1	**28**	0	**22**	95.2	**27**
Focused reassessments according to patient condition	20.3	**17**	5.17	**15**	21	**14**	20.8	**18**	5.17	**15**	88.8	**23**
Patient teaching about procedures, tests, and other diagnostic studies	25.3	**13**	5.17	**15**	24	**12**	13.5	**21**	5.17	**15**	79	**19**
Emotional support to patient and/or family	35.9	**8**	25.86	**3**	34	**6**	26	**14**	25.86	**3**	82.3	**20**

Teach patient about plans for their care after discharge and when to call after discharge	46.8	**4**	15.52	**6**	66	**2**	29.2	**11**	15.52	**6**	75.8	**16**
Attending family conferences/ interdisciplinary conferences	67.5	**2**	44.83	**1**	50	**3**	55.2	**1**	44.83	**1**	53.2	**6**
Ambulation 3 times per day or as ordered	66.2	**3**	31.03	**2**	93	**1**	53.1	**2**	31.03	**2**	66.7	**9**
Assist with toileting needs within 5 min of request	20.8	**16**	8.62	**10**	31	**7**	33.3	**10**	8.67	**9**	50	**4**
Response to call light is initiated within 5 min	37.8	**7**	5.17	**15**	28	**11**	42.7	**4**	5.17	**15**	70.9	**10**
Setting up meals for patients who can feed themselves	28.8	**11**	13.8	**7**	17	**17**	52.1	**3**	13.8	**7**	70.9	**10**
Full documentation of all necessary data	40.5	**6**	6.89	**14**	21	**14**	19.8	**19**	6.89	**14**	82.3	**20**

Sit up the patient out of bed	74	**1**	18.96	**5**	38	**4**	41.7	**5**	18.69	**5**	64.6	**8**
Use of preventive measures for patient at risk of falling	15.4	**20**	0	**22**	10	**21**	27.1	**13**	0	**22**	75.8	**16**
Airway aspiration	12.7	**23**	0	**22**	10	**21**	41.7	**5**	0	**22**	43.6	**2**
Feeding the patient or administering the diet by probe tube at the proper time	12.8	**22**	6.9	**12**	10	**21**	9.4	**23**	6.9	**12**	50	**4**
Hydrating the patient by providing oral fluids or by administering the probe tube	11.8	**24**	0	**22**	21	**14**	28.1	**12**	0	**22**	72.6	**13**

Emboldened – individual study rank ordering
