# Supplementary material for: Missed nursing care in acute care hospital settings in low-income and middle-income countries: a systematic review
Source: Hum Resour Health. 2023 Mar 14;21:19. doi: 10.1186/s12960-023-00807-7 (PMC10015781; doi:10.1186/s12960-023-00807-7)
Supplement: Supplementary file 5 — Additional file 5. Table showing ranked nurse self-reported reasons for missed nursing care using the MISSCARE instrument, median rank across primary studies and relative position of reasons for missed nursing care. (The median rank is the median of all individual study ranks across all studies, while the position compares the relative position of each reason based on the median rank). [file 12960_2023_807_MOESM5_ESM.pdf]

S5 Table: Table showing ranked nurse self-reported reasons for missed nursing care using the MISSCARE instrument, median rank across primary studies and relative position of reasons for missed nursing care.

(The median rank is the median of all individual study ranks across all studies while the position compares the relative position of each reason based on the median rank)

Category of reason	Reason	Al-Faouri et al*	Saqer et al*	Haftu et al*	Lima et al*	Lima Silva et al*	Moura et al*	Silva et al*	Median rank[$]	Position[%]
Communication	Tension or communication breakdowns within the nursing team	11	9	6	16	10	16	7	10	11th
	Lack of back up support from team members	6	9	15	15	12	17	14	14	16th
	Nursing assistant did not communicate that care was not done	13	17	2	14	5	14	7	13	13th
	The professional responsible for providing the care or caregiver was outside the unit/sector or was unavailable	17	16	16	16	16	15	13	16	17th
	Tension or communication breakdowns with the medical staff	8	9	7	13	17	13	7	9	9th

	Tension or communication breakdowns with other support departments	9	9	14	11	11	12	12	11	12th
	Other departments did not provide the care needed	12	13	13	8	15	10	14	13	13th
	Inadequate hand-off from previous shift or sending unit	15	13	17	11	10	10	16	13	13th
Material resources	Unbalanced patient assignments	7	7	12	10	12	9	7	9	9th
	Supplies/equipment not available when needed	9	4	1	7	9	2	7	7	6th
	Supplies/equipment did not function properly when needed	13	4	4	6	6	1	6	6	5th
	Medications were not available when needed	16	13	3	4	7	7	5	7	6th
**Labour resources**	**Unexpected rise in patient volume and/or acuity on the unit**	**2**	**2**	**4**	**3**	**3**	**5**	**17**	**3**	**2nd**
	Urgent patient situations (eg, a patient's condition worsening)	4	3	9	4	1	4	4	4	4th
	**Inadequate number of staff**	**1**	**1**	**9**	**1**	**1**	**5**	**1**	**1**	**1st**
	**Inadequate number of assistive personnel (e.g., nursing assistants, technicians, etc)**	**3**	**7**	**11**	**2**	**3**	**3**	**2**	**3**	**2nd**

	Heavy admission and discharge	5	4	8	9	8	8	3	8	8th

* -Values within these columns are individual study ranks for nurse self-reported reasons for missed nursing care.

$ -The calculated median rank across all individual studies

@ - The IQR is the interquartile range of individual study ranking across all studies

% - The position is the relative position of reasons for missed nursing care based on the calculated median rank across studies

The Emboldened rows are the top 3 nurse self-reported reasons for missed nursing care based on calculated median rank and relative positions of the reasons across all studies.
